# Double lysis: an integrative time-saving method yielding high-quality RNA from strawberry

**DOI:** 10.1186/s43141-020-00039-5

**Published:** 2020-06-24

**Authors:** Mohamed Hazman, Farida Kabil, Shrouk Abd Elhamid, Peter Nick

**Affiliations:** 1 Agricultural Genetic Engineering Research Institute (AGERI), Agricultural Research Center (ARC), 9 Gamma St, Giza, 12619 Egypt; 2 Vegetable Crops Department, Faculty of Agriculture, Cairo University, Gamma St., 12613, Giza, Egypt; 3 Molecular Cell Biology, Botanical Institute, Karlsruhe Institute of Technology, Bldg. 30.43, Fritz-Haber-Weg 4, 76131 Karlsruhe, Germany

**Keywords:** *Fragaria × ananassa*, Strawberry, RNA, Leaves, Fruits, Double lysis

## Abstract

**Background:**

The isolation of high-quality RNA from strawberry leaves and fruits is notoriously cumbersome. Both tissues are extremely rich in active secondary metabolites, as phenolics and pigments that inevitably perturb the isolation of RNA. Many protocols have been developed to address this problem. However, they are either costly, or time-consuming, in particular for high number of many plant samples. We describe here a new method with an easy-to-handle approach to obtain high-quality RNA from strawberry leaves and fruits. The method, referred to as double lysis, uses a novel combination of CTAB and Trizol protocols.

**Results:**

Compared to conventional Trizol-dependent protocols, either used individually, or in a commercial spin-column kit, the new method yields RNA at lower costs, but of significantly improved quality. The RNA obtained by double lysis was very pure as indicated by 260/280 ratios of 2.06 (leaves) and 2.07 (fruits), while 260/230 ratio was 2.07 and 1.75, respectively. Also visually, RNA sediments from double lysis showed a white color, indicative of efficient elimination of polyphenolics and pigments. In contrast, traditional Trizol method produced reddish precipitates. The purity of RNA isolated by double lysis enabled successful removal of genomic DNA and, thus, allowed for more efficient cDNA synthesis and RT-PCR. In addition, the suggested method is able to yield RNA with fully preserved integrity from strawberry leaves, fruits in addition to petals and roots.

**Conclusion:**

Double lysis is a new RNA isolation protocol developed through the integrative coupling of CTAB and Trizol reagents. The method is cost-effective, robust, time-saving, and can cope even with recalcitrant plant tissues with high contents of phenolics and pigments, such as strawberry leaves and fruits.

## Background

Cultivated strawberry (*Fragaria* × *ananassa* Duch.) is one of the most valuable fruit crops, but highly susceptible to both abiotic (such as salinity and drought) and biotic (such as fungal and viral infection) stress factors. To study stress-related gene expression, it requires to obtain high-quality RNA. In this context purity, integrity and quantity of the RNA are keys for successful molecular analysis by quantitative, semi-quantitative RT-PCR, or RNAseq [1].

Leaves of strawberry plants accumulate massive amounts of bioactive secondary metabolites including antioxidant compounds such as phenolic acids, ellagitannins, and flavonoids [2]. The profile of phenolic compounds depends on leaf age [3]. The same holds true for the abundance of phenolic compounds, which is highest in young leaves, even exceeding those of mature fruits [4]. Upon leaf lysis for RNA or DNA isolation, these phenolics are released from the fragmented cells and immediately oxidized to quinones that irreversibly bind RNA/DNA, deteriorating its quality and rendering it unsuitable for subsequent molecular studies [5].

Fully mature strawberry fruits are among the most difficult and challenging tissues in terms of achieving high-quality RNA [6]. The high abundance of cell-wall pectins accumulated during the maturation process, along with the high concentration of phenolic pigments, negatively influence isolation and purification efficiency, culminating in low recovery and difficulties to quantify the samples [7]

While several protocols allow to isolate RNA from strawberry leaves and fruits, these are usually very costly, since they rely on spin-column dependent, expensive kits, which is not an option for low-budget laboratories, in particular when a large number of samples has to be analyzed. The high price does by no means guarantee success, since even such expensive commercial kits can fail to cope with strawberry leaves or fruits [8, 9]. Extraction protocols based on CTAB and high-salt buffers can provide alternatives, but require extended incubation periods for proper RNA isolation [10]. Trizol, as further option, allows to isolate RNA from different plant tissues, and is well suited for rice leaves [11], but produced only limited success in case of strawberries [9, 12]. Therefore, we have developed a modified protocol integrating components from different extraction protocols, which allows for cost-effective extraction of high-quality RNA from strawberry fruits and leaves. In the current work, we describe and validate this method, termed double lysis, which might also be applied to obtain RNA with suitable quality for subsequent needed molecular reactions also from similarly pertinent tissues.

## Methods

### Plant materials

Fully extended leaves and ripe fruits were harvested from 3-month-old strawberry plants (*Fragaria* × *ananassa* Duch. cv Fertona) after transplanting. Both tissues were immediately shock-frozen in liquid nitrogen and then stored in -80°C for subsequent extraction of RNA and semi-quantitative RT-PCR.

### Buffers

Lysis buffer 1 [13]: 100 mM Tris-HCl, pH 8, 1.4% NaCl, 0.5% (v/v) Triton X-100 and 3% (w/v) CTAB (cetyl trimethyl-ammonium bromide). 2% (w/v) poly-vinyl pyrrolidone (PVP), and 5% (v/v) 2-mercaptoethanol (β-ME) were added just before use. Prewashing buffer (only in case of fruits) [14]: 100 mM Tris-HCl, pH 8, 350 mM sorbitol, 5 mM EDTA, pH 8.0 (the buffer should be stored at + 4 °C), in addition 3% (v/v) β-ME was added immediately before use.

### Reagents

Trizol (Ambion, Invitrogene, USA), chloroform:isoamylalcohol (24:1), isopropanol, 75% ethanol, absolute ethanol, and DNase-RNase free water, or double-distilled water.

### Kits

Direct-zol RNA miniprep (Zymo Research, USA).

RNA isolation via the double-lysis protocol:

- All centrifugation steps must be performed under cooling (+ 4 °C)
- Note: while the dissolution of the sediments requires several steps of short vortexing during preparation, never vortex the purified RNA, especially while dissolving it in water.

1. Weigh in maximally 0.03 g of leaf tissue or nearly 0.5 g of fruit tissue.

2. Grind the tissue in liquid nitrogen to fine powder using mortar and pestle.

- For fruits only, add 1 ml of prewashing sorbitol buffer (ice-cold) supplied with 30 μl β-ME for each sample, vortex thoroughly for 30 s, then spin down for 5 min at 3700×*g* on 4 °C. Completely discard the supernatant including any floating tissues. Repeat this step once more and then go to step 3.
- For leaves, go directly to step 3.

3. Add 1 ml of the lysis buffer (buffer 1) containing freshly added 50 μl of β-ME and 20 mg PVP. Resuspend the sediment by pipetting several times with cut tips till the tissue is resuspended completely, then vortex 30 s.

4. Incubate 10 min at 65 °C, invert the tubes gently a couple of times every 2 min (do not vortex during heat incubation) and centrifuge 5 min at 17,600×*g* on 4 °C.

5. Transfer 700 μl of the supernatant into a separate tube and add an equal volume of chloroform:isoamylalcohol (24:1). Vortex 10 s, then let settle at room temperature for 5 min. Spin down 15 min at 21,000×*g* on 4 °C.

6. Take the 400 μl supernatant and add 800 μl Trizol (ice cold), vortex 30 s, then let it at room temperature for 5 min.

7. Add 200 μl of chloroform, vortex 10 s, and incubate at room temperature for 5 min.

8. Spin down for 15 min at 21,000×*g* on 4 °C, and then transfer the upper 750 μl from the upper phase into a separate tube, where 500 μl isopropanol are added, and the mixture is then mixed by pipetting several times. Incubate the mixture at room temperature for 10–15 min, then transfer to – 20 °C for 10–15min.

9. Spin down for 15 min at 17,600×*g* on 4 °C, to obtain a visible precipitate and discard the supernatant.

10. Add 1 ml of 75% cold ethanol, then vortex 10 s. Re-spin for 3 min at 17,600×*g* and 4 °C. Discard the supernatant.

11. Repeat step 10.

12. Add 1 ml of absolute ethanol, vortex 10 s, then spin down for 5 min at 17,600×*g* on 4 °C.

13. Carefully discard the supernatant completely and let the precipitates to dry for 5 min only at room temperature. Do not lose the precipitate as at that stage it tends to be loose.

14. Add 30–50 μl of DNase-RNase free water (do not pipette or vortex) and keep in room temperature for 15 min then dissolve the white sediments carefully by gentle pipetting for few times. Note: never vortex at that stage, since it will disrupt RNA integrity.

### RNA isolation from strawberry petals and roots by double-lysis method

The protocol applied with leaves was also used to isolate RNA from petals (0.3 g) and roots (0.1 g) of strawberry plants, just omitting the prewashing step.

Other used protocols:

1. Trizol (Ambion, USA) according to the manufacturer instructions.
2. Direct-zol^TM^ RNA miniprep according to the manufacturer instructions.

### RNA quantification and purity level

The RNA was quantified spectroscopically (Nanodrop® Thermo Scientific, USA) at 260 nm. The level of purity was inferred from the ratios of *A*_260_/*A*_280_ for protein contamination, and *A*_260_/*A*_230_ for determining the contamination with polysaccharides and polyphenolics.

### DNase digestion

The isolated RNA of all samples was further digested with DNase using the product DNase I, RNase-free (Thermo Scientific, USA) according to the manufacturer instructions. The obtained RNA was directly used in subsequent cDNA synthesis reactions. It is recommended to not add more than 1 μl of DNase for more than 1 μg of isolated RNA, since this might affect RNA integrity.

### Reverse transcription-polymerase chain reaction (RT-PCR)

The cDNA was synthesized using the RevertAid First Strand cDNA synthesis kit (Thermo Scientific, USA). As template, 280 ng of RNA for at least three samples of leaves and fruits were added to 1 μl of oligo (dT)_18_ primers, mixed well, and heated at 65 °C for 5 min, before keeping the sample on ice for at least 2 min. Subsequently, the following mixture is added: 5× reaction buffer (1 μl), RiboLock RNase Inhibitor, 20 U/μL (1 μl), 10 mM dNTP Mix (2 μl), and RevertAid M-MuLV Reverse Transcriptase, 200 U/μl (1 μl). The whole mixture was incubated at 42 °C for 60 min and at 70 °C for 5 min to deactivate the reverse transcriptase and terminate the reaction. The mixture was then cooled on ice for at least 5 min and then diluted tenfold with 10 DEPC-treated water. Aliquots of 3 μl were used to amplify FaGAPDH2 (AF421493.1) encoding glyceraldhyde-3-phophate dehydrogenase using the forward primer 5′-CTTGAGAAGAAGGCCACCTATG-3′, and reverse primer 5′-CTTCGGTGTAACCCAAGATACC-3′. The product size should be 91 bp in case of purified RNA with no DNA contamination, but 200 bp with DNA template [15]. The PCR was run using the ready-to-use reaction mixture Cosmo PCR red master mix (Willowfort, UK) according to the manufacturer protocol using initial denaturation at 95 °C for 3 min, followed by 35 cycles of denaturation at 95 °C for 15 s, annealing at 58 °C for 30 s, and synthesis at 72 °C for 1 min. The final extension was conducted at 72 °C for 5 min. The reaction was stopped by pausing the reaction at 4 °C.

### Visualization of RNA or PCR products

The isolated RNA and the PCR amplicons were separated on an 1.5% agarose gel at 5 V/cm for 30 min and visualized with ethidium bromide along with a 100-bp size marker (Thermo Scientific, USA).

## Results

### Total RNA isolation by Direct-zol, Trizol, and double lysis

**Fig. 1:**
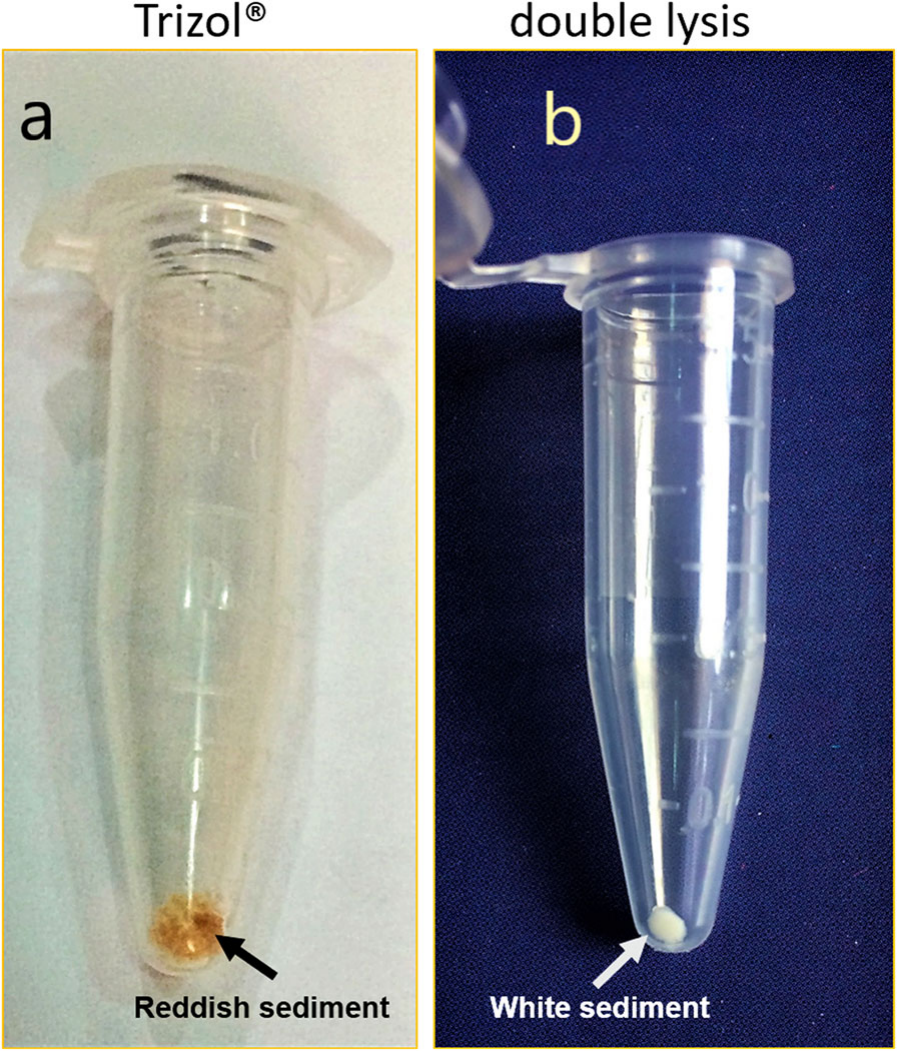
Variation in the appearance of RNA sediments isolated from strawberry leaves, **a** by standard Trizol-based extraction and **b** as compared to the double-lysis method

**Fig. 2:**
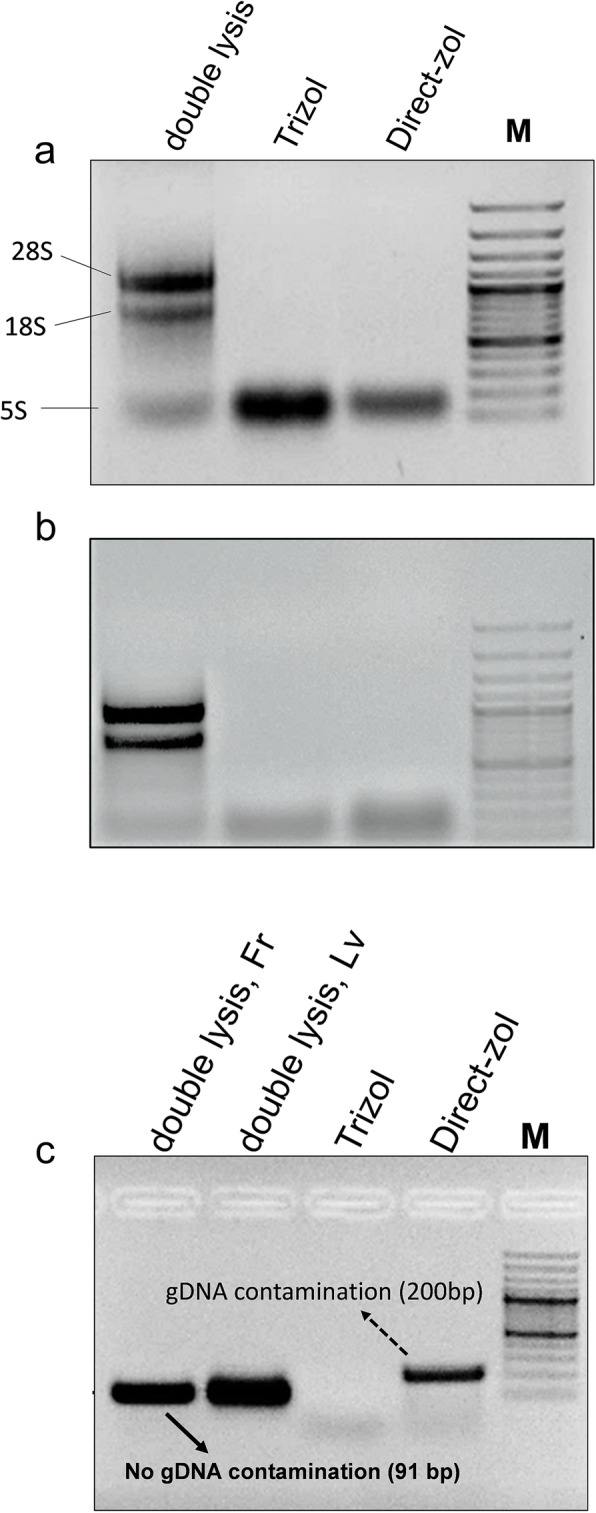
Integrity of RNA isolated from strawberry leaves **a** and fruits **b** assayed by electrophoretic separation using different extraction protocols: double-lysis method (left), conventional Trizol extraction and extraction by the Direct-zol kit. **c** Agarose gel electrophoresis showing the migration of RT-PCR product of a fragment of FaGAPDH2 spanning an intron. The expected size of the product is 200 bp in case of a genomic DNA template, and 91 bp in case of a cDNA template, either Direct-zol or Trizol samples are from leaves RNA. Templates obtained from RNA extracted by the double-lysis method from fruits (Fr) and leaves (Lv) are compared to templates from standard Trizol extraction or the spin-column based Direct-zol kit. Gene Ruler^TM^ 100 bp plus DNA ladder as M

The total RNA was planned to be isolated from strawberry leaves and fruits using three different isolation protocols: Direct-zol (Trizol-dependent spin column commercial kit), conventional Trizol method, and the double-lysis method that has been proposed in this work. During the process of isolation, the RNA sediments were observed to be larger in size but apparently reddish in color (Fig. 1a) while double-lysis method yielded white-colored RNA precipitate (Fig. 1b). Isolated RNA by the three different methods was altogether compared by gel electrophoresis in case of strawberry leaves (Fig. 2a) and ripe red strawberry fruits (Fig. 2b). The results indicated that double-lysis method could produce the full three 28S, 18S, and 5S ribosomal RNA bands while either Trizol or the commercial kit Direct-zol was only capable of obtaining the small 5S rRNA in both of leaves and fruits tissues. Furthermore, similar three intact bands could be successfully seen with RNA isolated from strawberry roots and petals tissues by double-lysis method. The yield of isolated RNA was spectroscopically quantified using Nanodrop® at 260 nm (Table 1).

**Table 1:** Yields of RNA isolated from strawberry leaves and fruits using three dependent methods

Tissue	Leaf (μg/g FW)		Fruit (μg/g FW)	
Method				
Direct-zol kit	25.00^b^	± 5.20	10.13	± 0.63
Trizol	794.93^a^	± 101.60	8.42	± 2.44
Double lysis	96.71^b^	± 7.29	8.57	± 0.89

With leaves, Trizol method could yield the highest concentration (794.93 μg/g FW) comparing to double lysis and Direct-zol kit, 96.7 and 25 μg/g FW, respectively. Nevertheless, the ratio of *A*_260_/_280_ (Fig. 4a) and *A*_260_/_230_ (Fig. 4b) indicated that RNA isolated by double-lysis method bears the highest quality comparing to Direct-zol and Trizol methods. The ratio *A*_260_/_280_ was found to be 1.95, 1.29, and 2.05 while *A*_260_/_230_ was 1.28, 0.58, and 2.07 for Direct-zol, Trizol, and double-lysis method, respectively. For red ripe strawberry fruit tissue, there was no significant difference in RNA yield among the three different used methods (Table 1). For RNA purity, double lysis showed superiority over Direct-zol or Trizol where *A*_260_/_280_ ratio was 2.09, 1.3, and 1.48 while *A*_260_/_230_ was 1.75, 0.45, and 0.49, respectively (Fig. 4a, b).

### Reverse transcription-polymerase chain reaction (RT-PCR)

In order to investigate the suitability of the isolated RNA with double-lysis method for sensitive molecular studies, RT-PCR test was accomplished using a specific pair of primers for FaGAPDH2 [15]. As shown in Fig. 2c, a PCR product of 91 bp was successfully amplified from cDNA generated from double-lysis isolated RNA in either strawberry leaves and fruits. On the other hand, Direct-zol produced a PCR product with undesired size (200 bp) while the cDNA generated from Trizol RNA failed to produce any PCR DNA band.

## Discussion

In this study, we have used three different RNA isolation protocols: either Trizol or Direct-zol, which is a Trizol-dependent kit (Zymo Research, USA), or a new integrative method, referred to as double lysis, using strawberry leaves and fully matured, red, and soft fruits. The RNA precipitates obtained by the Trizol and the double-lysis method differed with respect to amount and color (Fig. 1a, b). Unlike the completely white precipitates obtained from the double-lysis method (Fig. 1b), conventional Trizol-extraction produced more voluminous, yet dark reddishly colored sediments, indicative of co-precipitated polyphenols [16, 17]. The integrity of isolated RNA from strawberry leaves was then visualized by gel electorphoresis on a 1.5% agarose gel (Fig. 2a). While both Trizol-based extraction protocols (conventional extraction, as well as the Direct-zol kit, involving spin-columns) yielded only the small 5S ribosomal RNA [10], the double lysis was able to generate the full set of ribosomal RNA (28S, 18S, and 5S), reflecting that RNA integrity had been preserved. When the purity of the RNA was assessed spectrophotometrically by determining the *A*_260_/*A*_280_ (Fig. 4a), and the *A*_260_/*A*_230_ ratios (Fig. 4b), the double lysis was found to be superior to both Trizol-based extraction methods (albeit these methods yielded more RNA, Table 1). The *A*_260_/*A*_230_ ratio indicated that less polyphenols were co-precipitating with the RNA. Thus, the Trizol-based protocols, while yielding higher amounts of RNA (Table 1), produced RNA which was lower in purity and integrity compared to the double-lysis method, which yielded satisfying amounts of RNA (96.7 μg/g FW, Table 1), but with much higher quality with leaves (2.06 for *A*_260_/*A*_280_ ratio and 2.07 for *A*_260_/*A*_230_ ratio) and integrity.

**Fig. 3:**
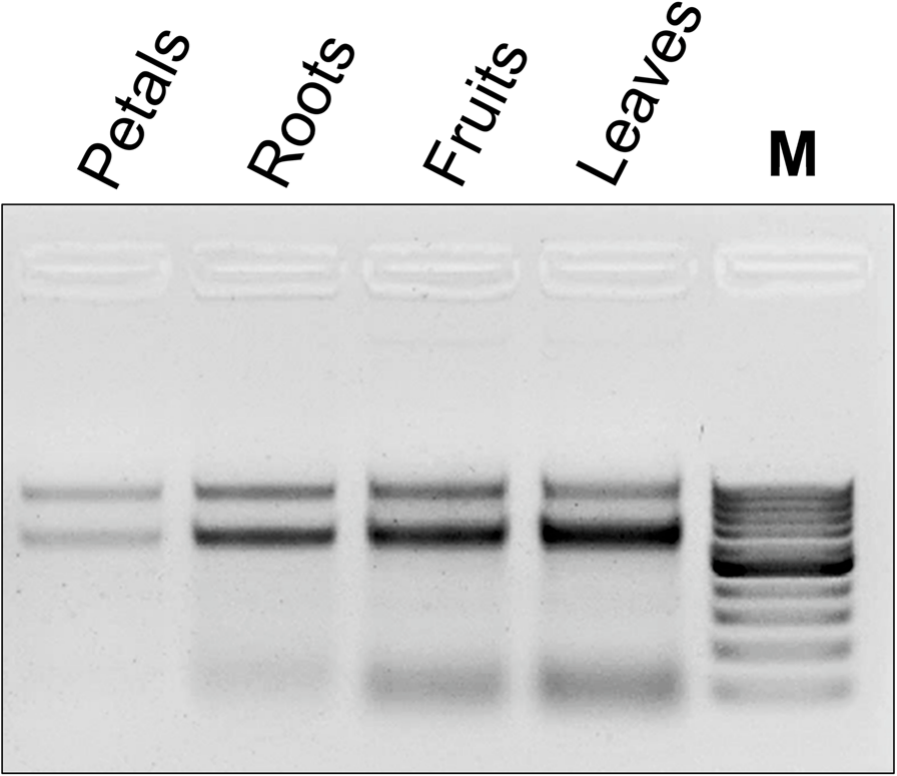
Total RNA isolation from leaves, fruits, roots, and petals of strawberry plants using double-lysis method. M Gene Ruler ^TM^ 100 bp DNA marker

**Fig. 4:**
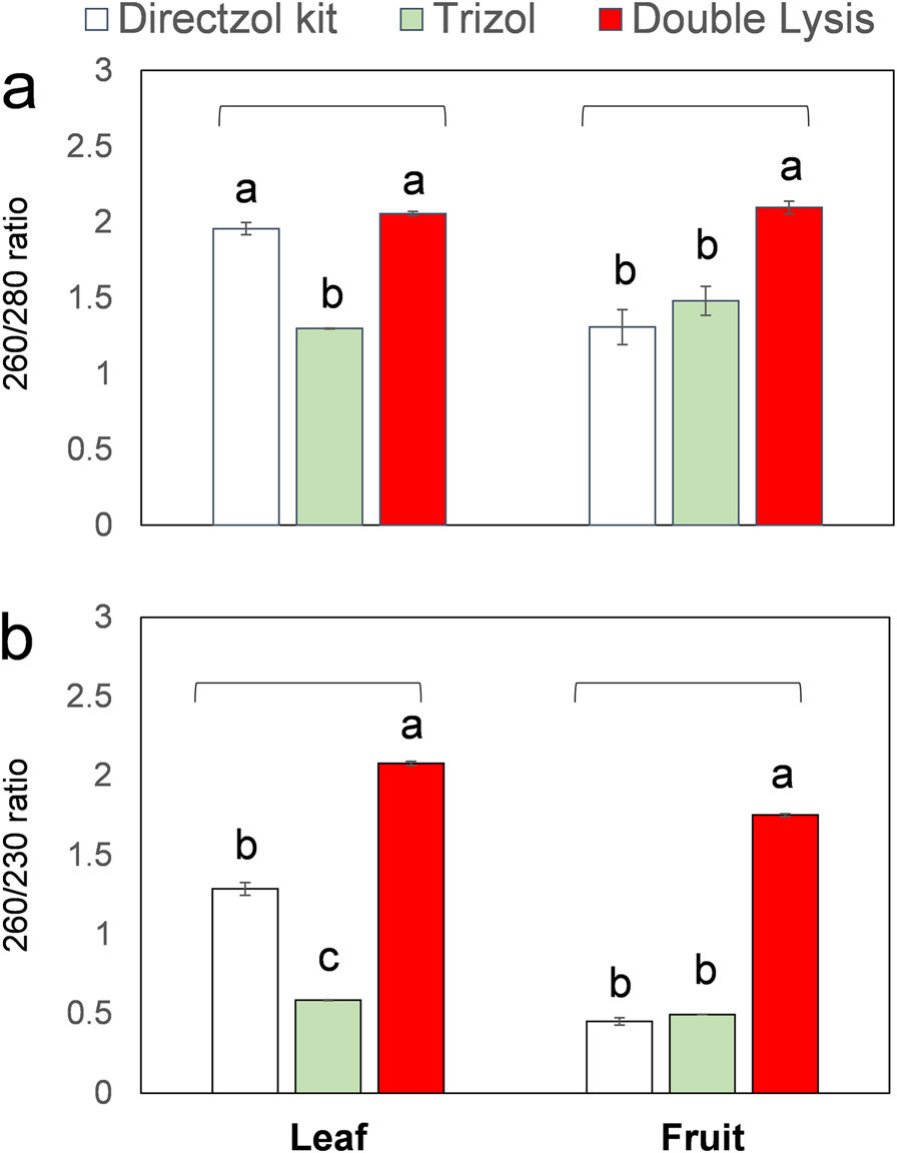
Purity of RNA isolated from strawberry leaves (left) and fruits (right) assayed spectrophotometrically. **a***A*_260_/*A*_280_ ratio **b***A*_260_/*A*_230_ ratio. Values represent means ± SE of three independent biological replications. Means with the same letters are not significantly different according to Tukey’s honest significant (HSD) test (*p* ≤ 0.05)

Compared to leaves, fruit tissue of strawberries is even more challenging, because it is soft with high water content, pectin-rich cell walls, and fully expanded vacuoles, such that the genetic material including RNA is diluted by a chemically adverse environment [18]. To compensate for the lower ratio of nuclear to total cell volume, sample weight has to be increased from 0.03 g (as in case of the leaves) to 0.5 g soft fruit tissue. The fine powder resulting from grinding is voluminous, of pink color, and fills almost half of the standard reaction tube (1.5 ml). The anthocyanins released from the broken vacuoles and responsible for the color, are highly problematic for RNA extraction, because they can sequester nuclear acids. We have therefore adopted a prewashing step which was originally designed for DNA isolation from leaves of *Dimorphandra mollis* [14]. This prewashing step with ice-cold sorbitol buffer (see “Methods” section) was found to be very useful in removing anthocyanin from strawberry fruits; this might also concern other recalcitrant plants such as *Lafoensia* spp. [19]. After this step, the remaining sediment should appear white or at least pale pinkish. The RNA isolated this way was of good integrity, as indicated by the fact that all three rRNA bands (28S, 18S, and 5S) were clearly visible after gel electrophoresis, while the two other methods, where Trizol was used, failed to produce any of the two higher RNA bands (Fig. 2b). While the amount of RNA isolated by the double-lysis method was not significantly different from the two Trizol-based protocols (Table 1), it produced RNA of superior purity as indicated by *A*_260_/*A*_280_ and *A*_260_/*A*_230_ ratios of 2.1 and 1.75, respectively (Fig. 4a, b). Additionally, the double-lysis method was successful in obtaining three intact RNA bands for 28S, 18S, and 5S RNA in case of strawberry roots and petals (Fig. 3).

To further assess the quality of the purified RNA, we amplified a diagnostic fragment from FaGAPDH2 [16] by RT-PCR. To avoid false-positive amplificates caused by contaminations with genomic DNA, the RNA was digested with DNase. The fragment was selected to span an intron, such that the amplicon to be expected from pure cDNA should have a size of 91 bp, while in case of contamination by genomic DNA, a band of 200 bp should result. Indeed, for both, leaves and fruits, the double-lysis method led to a single, clear amplicon with a size of 91 bp, indicating that the RNA template was free from genomic DNA. In contrast, the conventional Trizol extraction did not lead to any band, and the PCR product from cDNA deriving from a template extracted by the Direct-zol kit was 200 bp in length, indicating contamination by genomic DNA that was even exceeding the cDNA template, since the smaller band with 91 bp was not detectable (Fig. 2c). This gDNA contamination in case of Direct-zol kit was indicative of insufficient DNA digestion. The very low RNA yield (25 μg/g FW) obtained from strawberry leaves by Direct-zol kit made it technically difficult to adjust DNase concentration (not more than 1 U of DNase I, RNase-free per 1 μg of RNA should be used to avoid cross-digestion of RNA itself), such that the range between incomplete digestion of the contaminating gDNA, and RNA damage is very narrow, introducing unpredictable variability. Therefore, we recommend using on-column DNase for digesting possible co-isolated DNA in case of using spin-column RNA isolation kits. The failure to amplify the correct amplicons from Trizol-based extraction is very likely caused by the low quality of isolated RNA (Figs. 1 and 4), probably due to the contamination with polyphenoles and polysaccharides [13].

## Conclusion

**Fig. 5:**
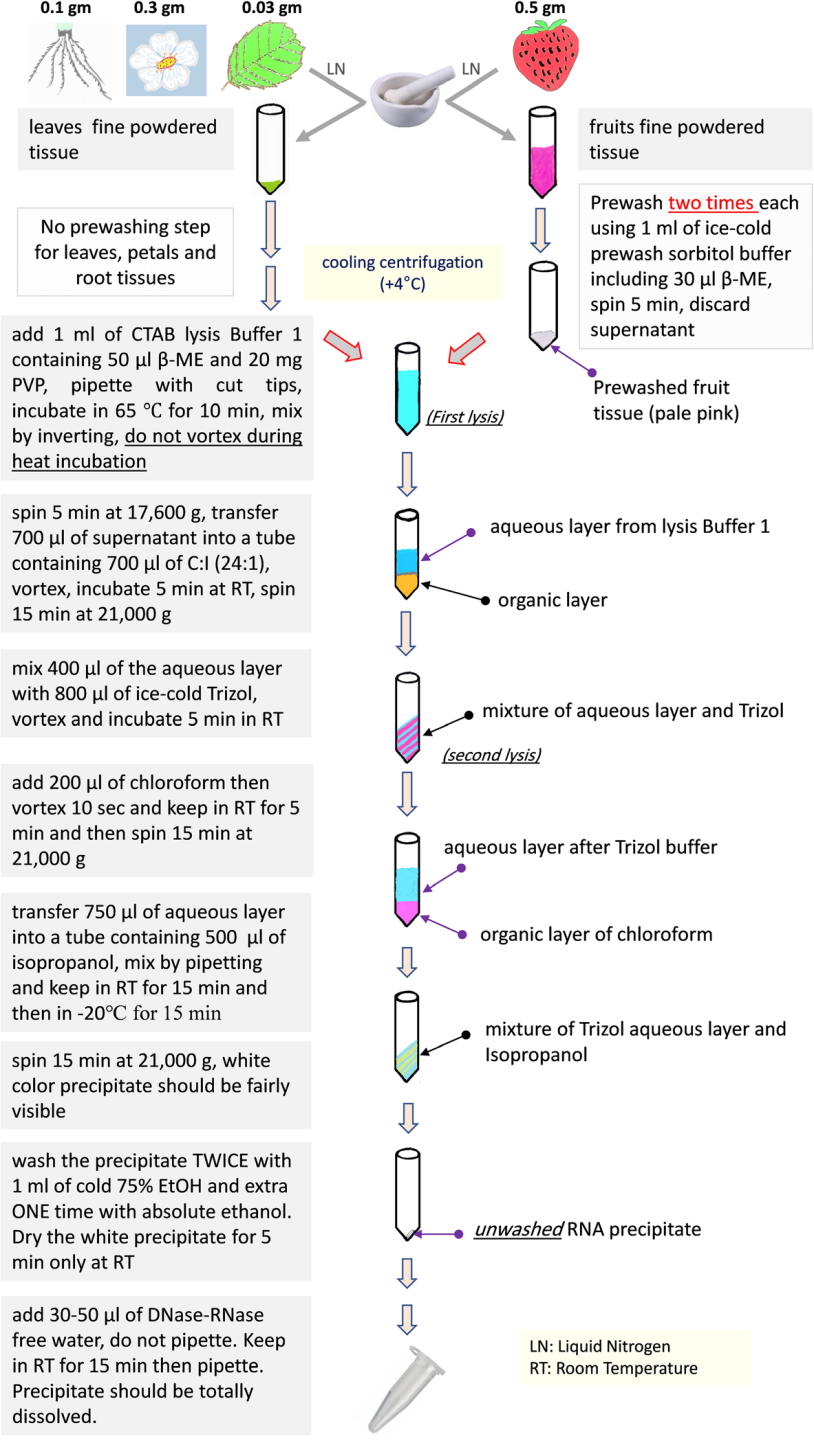
Schematic flow diagram for isolating RNA from strawberry leaves, fruits, petals, and roots using double-lysis method

The critical issue for RNA isolation from challenging tissues, such as strawberry leaves and/or fruits, is to minimize contamination by polysaccharides and polyphenols rather than the quantity. Our method addresses this issue in a simple, robust, cost-effective, and time-saving way and even can cope with strawberry leaves and fruits as particularly challenging material to yield RNA in satisfying quantity, yet in high quality (see Fig. 5 for detailed schematic flowchart). The main novelty is the integration of a CTAB-based lysis step. The new double-lysis method performed superior to conventional Trizol-based extraction that yielded only heavily contaminated RNA with a very poor level of integrity. A crucial point for the success of RNA extraction from fruit tissue was the pre-washing step to remove anthocyanin pigments and an increase of sample weight to compensate for the large vacuoles in those cells. While this protocol was developed for strawberry leaves and fruits, it has the potential to be widely applied to other plant materials that are recalcitrant due to abundance of secondary metabolites contents or low RNA content. We plan to test this, for instance, for woody tissues.

## Data Availability

The datasets used and/or analyzed during the current study are available from the corresponding author on reasonable request.

## Abbreviations

cDNA: Complementary DNA
CTAB: Cetyl trimethylammonium bromide
EDTA: Ethylenediaminetetraacetic acid
FaGAPDH2: Glyceraldhyde-3-phophate dehydrogenase 2
PVP: Polyvinylpyrrolidone
RNA: Ribonucleic acid
RT-PCR: Reverse transcription-polymerase chain reaction
β-ME: 2-mercaptoethanol
